# The Combination of X-Ray Crystallography and Cryo-Electron Microscopy Provides Insight into the Overall Architecture of the Dodecameric Rvb1/Rvb2 Complex

**DOI:** 10.1371/journal.pone.0146457

**Published:** 2016-01-08

**Authors:** Noella Silva-Martin, María I. Daudén, Sebastian Glatt, Niklas A. Hoffmann, Panagiotis Kastritis, Peer Bork, Martin Beck, Christoph W. Müller

**Affiliations:** Structural and Computational Biology Unit, European Molecular Biology Laboratory (EMBL), Heidelberg, Germany; Centro Nacional de Biotecnologia (CNB-CSIC), SPAIN

## Abstract

The Rvb1/Rvb2 complex is an essential component of many cellular pathways. The Rvb1/Rvb2 complex forms a dodecameric assembly where six copies of each subunit form two heterohexameric rings. However, due to conformational variability, the way the two rings pack together is still not fully understood. Here, we present the crystal structure and two cryo-electron microscopy reconstructions of the dodecameric, full-length Rvb1/Rvb2 complex, all showing that the interaction between the two heterohexameric rings is mediated through the Rvb1/Rvb2-specific domain II. Two conformations of the Rvb1/Rvb2 dodecamer are present in solution: a stretched conformation also present in the crystal, and a compact conformation. Novel asymmetric features observed in the reconstruction of the compact conformation provide additional insight into the plasticity of the Rvb1/Rvb2 complex.

## Introduction

Rvb1 and Rvb2 (also known as RuvBL1/RuvBL2, Pontin/Reptin, TIP49a/TIP49b) are necessary for cell viability and play essential roles in various complexes involved in fundamental processes such as transcription regulation, DNA damage response and apoptosis (via the chromatin remodelling complexes SWR1, INO80 and TIP60), maturation of small nuclear ribonucleoproteins, cellular development, cancer metastasis and regulation of mitosis [1]. Rvb1 and Rvb2 are highly conserved eukaryotic proteins, very similar to each other, that belong to the large AAA+ (ATPases associated with diverse cellular activities) superfamily of ATPases [2]. Both proteins contain ATP binding and hydrolysis motifs located within two structurally defined domains [3]. Domain I (DI) contains the Walker A and B, arginine finger and sensor I motifs, while domain III (DIII) contains the sensor II motif. Together these two domains represent the AAA+ core and are sufficient to form hexameric rings [4]. Domain II (DII) corresponds to an insertion of 160–170 amino acids in domain DI between the Walker A and B motifs that is unique to Rvb1 and Rvb2 in comparison with other AAA+ family members [3]. DII adopts an oligonucleotide-binding fold (OB-fold) attached to the core by a flexible linker formed by two antiparallel β-strands [5]. It has been proposed that DII is important for DNA/RNA binding [3], regulating the helicase activity [4] and dodecamer assembly [4,6,7].

The different activities of the Rvb1/Rvb2 complex are not only regulated by cofactors, but also by its multiple assembly states. The INO80 complex that exchanges histone variant H2A.Z with H2A contains the Rvb1/Rvb2 dodecamer as nucleosome-interacting module [8]. The SWR1 complex that carries out the reverse reaction only contains one Rbv1/Rvb2 hetero-hexamer [9]. The structural plasticity of the Rvb1/Rvb2 complex appears to correlate with its various activities. Its ATPase activity is enhanced in Rvb1/Rvb2 dodecamers [6], while its helicase activity seems to increase upon deletion of the DII domains in the dodecamers [4]. Moreover, nucleosome binding has been shown for homo-hexamers of Rvb1 and Rvb2 [10], and also for Rvb1/Rvb2 dodecamers in the INO80 complex, but not for isolated Rvb1/Rvb2 dodecamers [8].

The different oligomeric states of the Rvb1/Rvb2 complex have therefore been the subject of numerous structural studies [4,7,11–16]. Crystal structures of human Rvb1 [3] and a truncated version of Rvb2 lacking DII [5] reveal similar homo-hexameric arrangements. The DII domain has been also truncated in the crystal structure of the human Rvb1/Rvb2 complex [4] that shows a dodecameric arrangement of the two heterohexameric rings stacked on top of each other. Moreover, all negative-stain and cryo-electron microscopy (EM) reconstructions show dodecameric complexes with interactions between the hexameric rings mediated by the DII domains [7,12–16]. Conformational flexibility of DII has been also characterized by molecular dynamics simulations [5,17]. Several EM studies suggest compact or stretched conformations or even a continuum of conformations that differ in the orientation of the DII domains in the middle region [7,13]. Nevertheless, the way the two rings interact in the dodecamer and how this results in different conformations is still not known in molecular detail.

Here, we report the crystal structure of the dodecameric Rvb1/Rvb2 complex from *Chaetomium thermophilum (Ct)* at 2.9 and 3 Å resolution in two nucleotide binding states (ADP/ADP and ADP/PP) together with two cryo-EM reconstructions of the same complex in the stretched and compact conformation at 21 and 12 Å resolution. By combining the structural information that has been obtained by these two independent techniques, we provide detailed, molecular information of the Rvb1/Rvb2 dodecamer obtained by X-ray crystallography with low resolution information of the global plasticity of the complex obtained by cryo-electron microscopy.

During the preparation of this manuscript, another crystal structure of the dodecameric Rvb1/Rvb2 complex from *Chaetomium thermophilum* has been published in the ADP/ADP and ATP/apo forms [18]. The four crystal structures are very similar with r.m.s. deviations varying between 0.87 Å to 1.14 Å resolution. Complementary SAXS experiments presented in the other study [18] suggested also conformational heterogeneity of the Rvb1/Rvb2 dodecamer in solution.

## Results and Discussion

### *Ct* Rvb1 and *Ct* Rvb2 form stable hetero-dodecamers in solution

**Fig 1:**
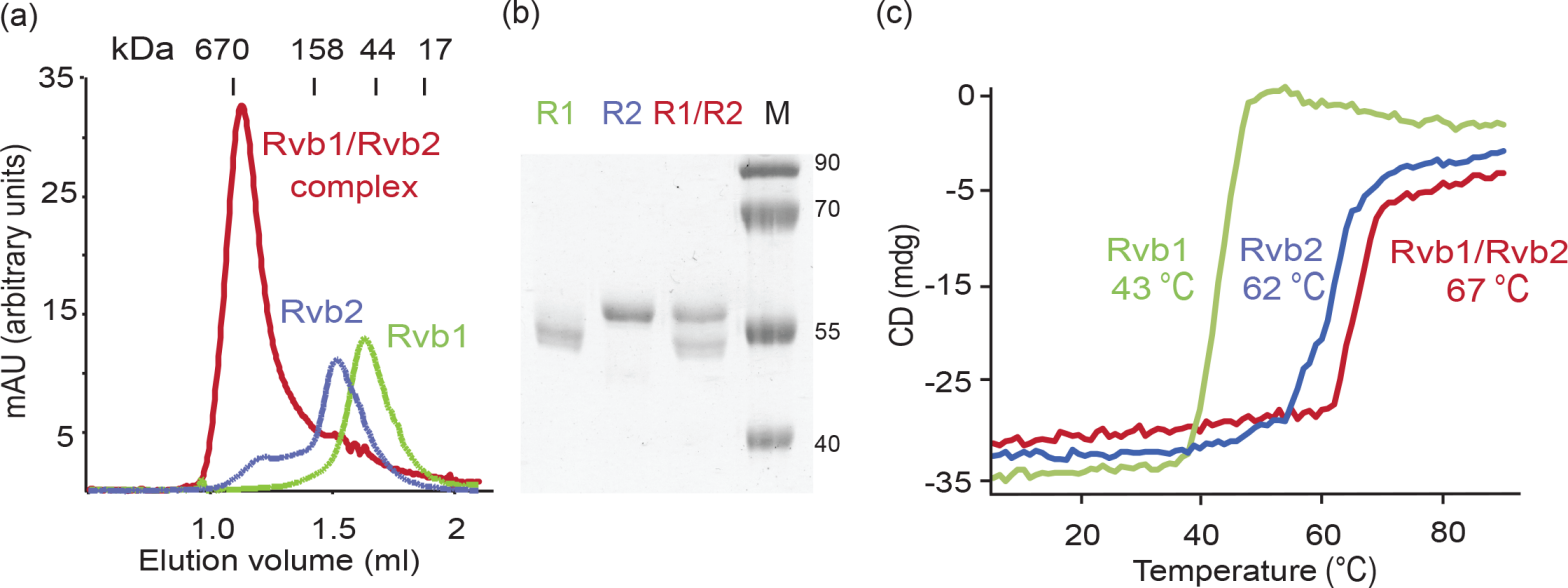
Rvb1/Rvb2 dodecamer purification and thermostability assays. (a) SEC profile of the purification of the individual proteins Rvb1 (green line) and Rvb2 (blue line), and the Rvb1/Rvb2 complex (red line). Molecular weight standards corresponding to 670, 158, 44 and 17 kDa are shown on top. (b) SDS-PAGE gel showing the proteins present in the peaks of the SEC shown in (a). (c) Denaturation curves of Rvb1, Rvb2 and Rvb1/Rvb2 with the same colour code as in (a).

We used the eukaryotic fungus *Chaetomium thermophilum* (*Ct)* for structural studies as the increased thermal stability of its proteins is advantageous for the structural characterization of dynamic complexes such as the Rvb1/Rvb2 complex [19–21]. The orthologous *Rvb1* and *Rvb2* genes were identified due to their high sequence conservation across the eukaryotic kingdom. *Ct* Rvb1 shares 68% and 71% sequence identity with human and *S*. *cerevisiae* Rvb1, respectively, while *Ct* Rvb2 shares 68% and 73% sequence identity with human and *S*. *cerevisiae* Rvb2, respectively (S1 Fig). We separately expressed Rvb1 and Rvb2 in *E*. *coli* and purified them to homogeneity. Subsequently, the Rvb1/Rvb2 complex was reconstituted from individually purified proteins by mixing them at a 1:1 ratio. Both individual proteins showed uniform peaks on size exclusion chromatography (SEC) corresponding to the predicted molecular weight of ~50 kDa for the monomers (Fig 1A). The Rvb1/Rvb2 complex also eluted as a single species, corresponding to a dodecameric form with ~620 kDa, where Rvb1 and Rvb2 are both present at stoichiometric quantities (Fig 1B). The Rvb2 profile shows a small peak that might indicate the presence of oligomers in the sample. However, negative stain electron microscopy (EM) grids of purified Rvb2 showed that it is monomeric under these conditions (S2A Fig). In contrast, previous purifications of the Rvb1/Rvb2 complex from other organisms reported a mixture of hexamers and dodecamers [7,13], pointing to the higher efficiency of dodecamer formation in *Chaetomium thermophilum* (S2B Fig). The dodecameric Rvb1/Rvb2 complex shows increased thermostability in comparison to the individual proteins: 43°C for Rvb1, 62°C for Rvb2 and 67°C for the complex (Fig 1C).

### Crystal structure of the Rvb1/Rvb2 complex and inter-ring interactions

**Fig 2:**
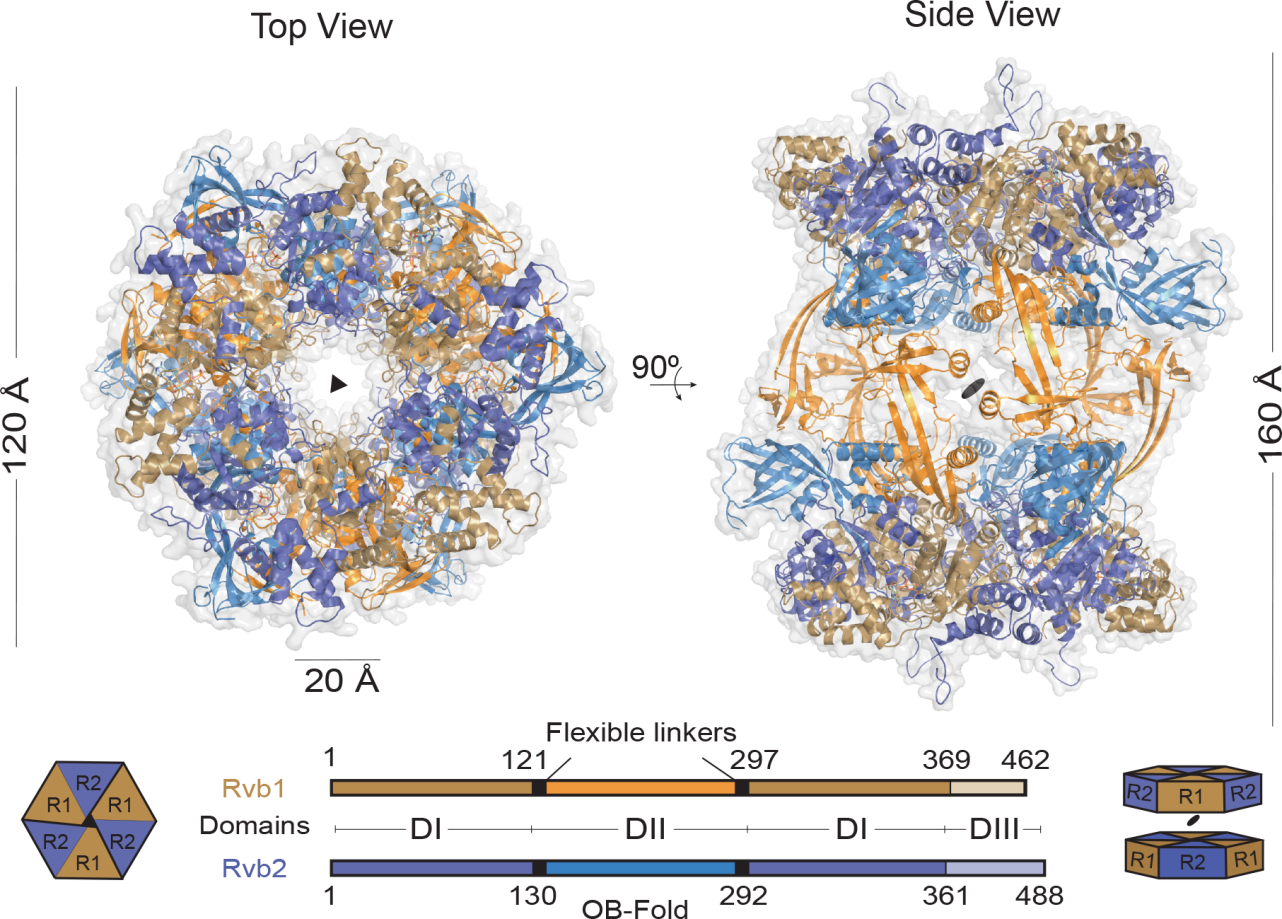
X-Ray structure of the Rvb1/Rvb2 dodecamer. Ribbon representation of the Rvb1/Rvb2 dodecamer in top and side views. Rvb1 and Rvb2 proteins are depicted in gold and blue, respectively. The DII domains are highlighted in orange for Rvb1 and bright blue for Rvb2. The relative orientation of the rings is illustrated by the cartoons. The schematic representation of the domain architecture of Rvb1 and Rvb2 is depicted below (DI, DII, DIII, OB-fold and flexible linker).

Crystals of the Rvb1/Rvb2 complex diffracting to 3 Å resolution were obtained by the vapour diffusion method after 5 to 10 days. Our attempts to correctly place individual molecules by molecular replacement using human RuvBL1 (PDB code 2C90) and human RuvBL2 (PDB code 2XSZ) failed due to the impossibility to distinguish Rvb1 from Rvb2 at the given resolution. Therefore, the complex structure was solved using selenomethionine (SeMet) substituted proteins in a single anomalous dispersion (SAD) experiment and subsequently refined to R_work_/R_free_ values of 23.0/25.6% (S1 Table and S3 Fig). The Rvb1/Rvb2 complex crystallized in space group H32 with one Rvb1/Rvb2 heterodimer in the asymmetric unit. Applying the crystallographic 3-fold axis generates hetero-hexamers with alternating Rvb1 and Rvb2 molecules, while adjacent hetero-hexamers are related by a crystallographic dyad to produce the full dodecameric Rvb1/Rvb2 assembly (Fig 2). The Rvb1/Rvb2 complex resembles a barrel with ~160 Å height, ~120 Å outer diameter and a central channel of ~20 Å diameter that would allow the passage of single-stranded DNA [3,7]. In this arrangement the hetero-hexameric rings are rotated with respect to each other by 60 degree, hence Rvb1 and Rvb2 are stacked on top of each other. The relative positions of the rings is therefore different to the one observed for the DII-deleted human Rvb1/Rvb2 dodecamer where both hetero-hexameric rings perfectly align [4].

**Fig 3:**
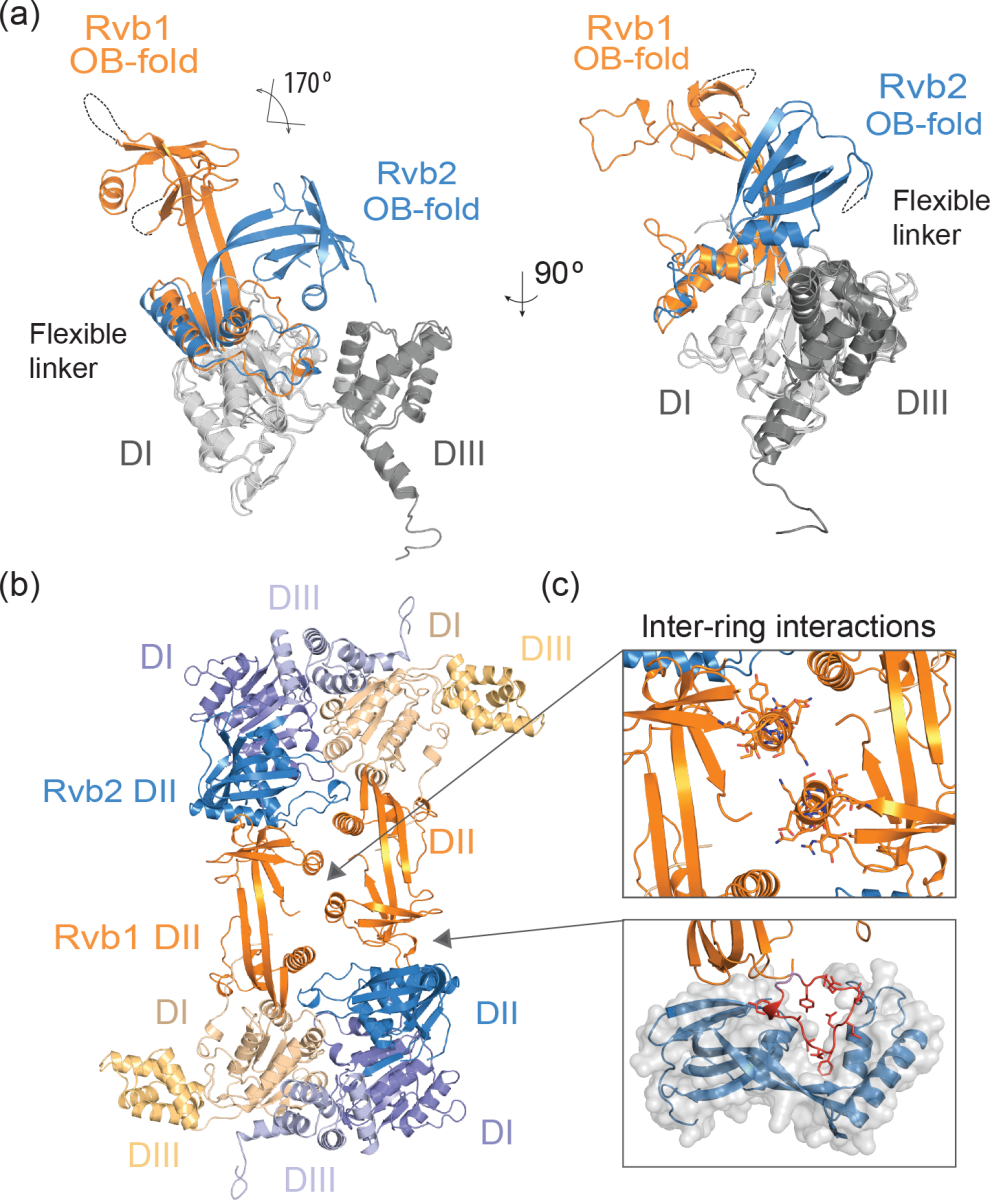
Detailed view of the DII domains and the inter-ring interface. (a) Superimposition of the core domains of Rvb1 and Rvb2 illustrating the different orientations of the DII domains that are related by ~170 degrees. (b) Side view showing the interface between two adjacent hexamers. (c) Structural details of the inter-ring interactions: DII of Rvb1 in the top ring interacts with DII of Rvb1 in the bottom ring (upper panel) and DII of Rvb1 (orange ribbons, the “inter-loop” is shown in red) interacts with the DII of Rvb2 (blue ribbons and grey surface) in the lower panel.

Consistent with previous EM reconstructions [7,12–16] and crosslinking data [8], the interaction between both hetero-hexameric rings is mediated by the DII domains, and notably the crystal structure of the *Ct* Rvb1/Rvb2 complex shows these interactions in greater detail. While the domains DI and DIII of Rvb1 and Rvb2 superimpose very well (r.m.s.d._550Cα_ = 1.09 Å), the domains DII of Rvb1 and Rvb2 are tilted in opposite directions (Fig 3A). Due to the flexibility of the two antiparallel β-strands in the linker, the OB-folds present in the Rvb1 and Rvb2 are rotated by ~170 degrees with respect to each other. In Rvb1 the linker adopts a straight conformation with the OB-fold pointing away from the AAA+ core in a similar conformation to the human Rvb1 homohexamer [3], while in Rvb2 the linker is bent with the OB-fold packing against the AAA+ core. The position of the DII domain in Rvb2 agrees with the prediction based on molecular dynamics simulations [17]. The inter-ring interactions in the *Ct* Rvb1/Rvb2 dodecamer are mainly mediated by the DII domains of Rvb1 (Fig 3B), in agreement with results from SEC and analytical ultracentrifugation studies [6]. The DII of Rvb1 from the top ring interacts with the symmetry-related DII of Rvb1 from the bottom ring through the only α-helix (residues 173 to 185) of the OB-fold (Fig 3C). A second interaction involves a linear motif (“inter-loop”, residues 208 to 221) of Rvb1 that packs against the β-strands and the two first helices of the DII of Rvb2 (Fig 3C). Hence, the described interface requires two distinct conformations of the DII domains from Rvb1 and Rvb2 to form the dodecameric complex.

### Nucleotide binding pockets in the Rvb1:ADP/Rvb2:ADP and Rvb1:ADP/ Rvb2:PP complexes

**Fig 4:**
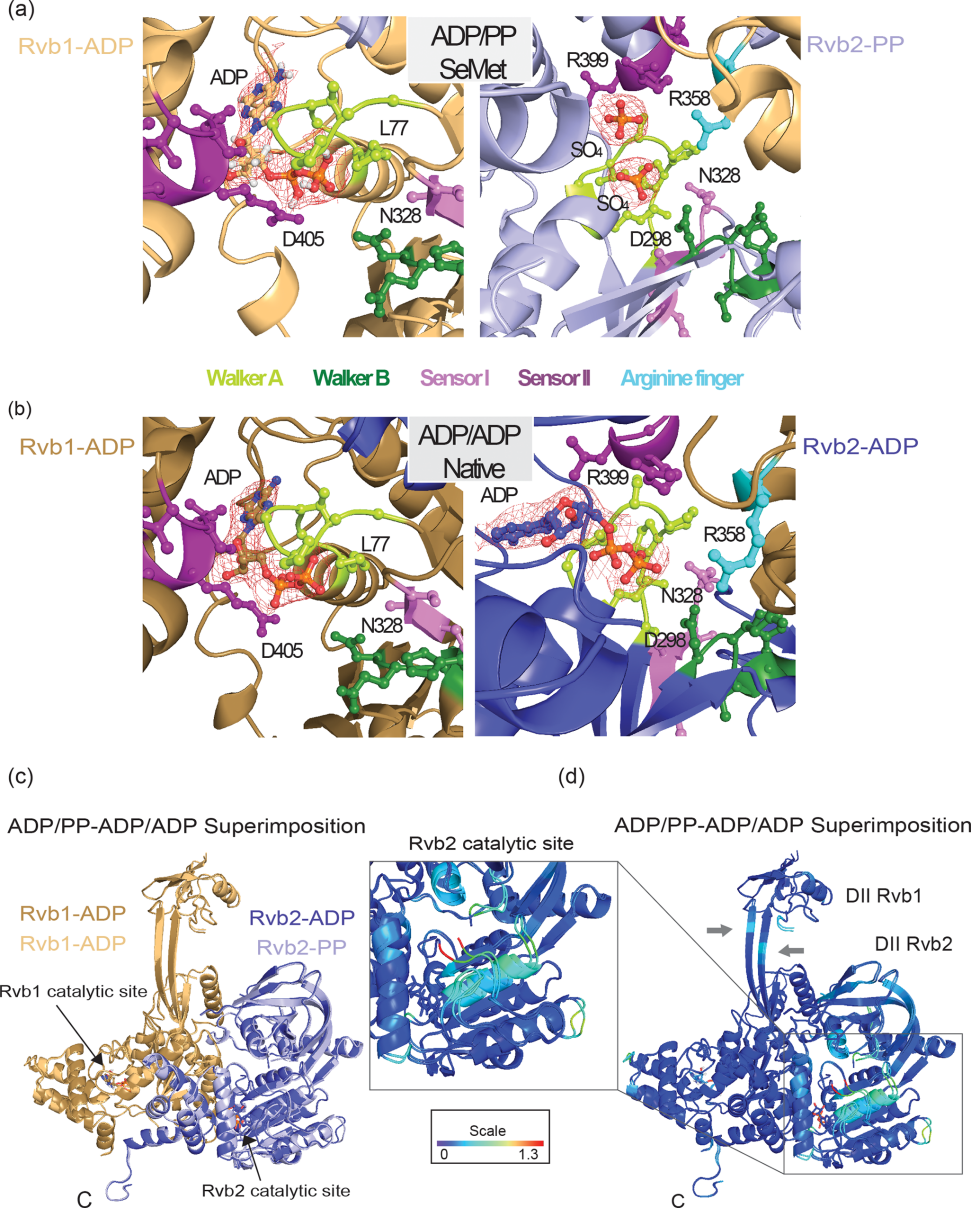
Nucleotide binding pockets in the Rvb1/Rvb2 dimers. Nucleotide binding pocket of the SeMet ADP/PP dimer (a) and the Native ADP/ADP dimer (b). Rvb1 is shown in the left and Rvb2 in the right, catalytic motifs are colour coded as stated below (a). The experimental density of the nucleotides is depicted as mesh. (c) Superimposition of the ADP/PP and ADP/ADP dimers of Rvb1/Rvb2. Rvb1 is depicted in light gold and Rvb2 in light blue in the ADP/PP dimer, while Rvb1 is shown in gold and Rvb2 in blue in the ADP/ADP dimer. (d) The maximum likelihood superimposition shown in (c) is displayed using B-Factor colouring highlighting the dynamic regions around the Rvb2 catalytic site (inset) and in the flexible linkers of Rvb1 and Rvb2 (grey arrows).

The Rvb1/Rvb2 crystals were obtained in the presence of ADP that was included during purification and co-crystallization. The SeMet crystals revealed density in Rvb1 that could be identified as ADP, whereas Rvb2 clearly shows two density peaks at the same position most likely corresponding to sulphates (Fig 4A) present in the crystallization solution and known to mimic phosphates. We will therefore refer to this state as ADP/PP. In the crystal structure of the native protein (solved using the SeMet map as a starting model) we observed very similar densities in Rvb1 and Rvb2 that could be identified as ADP molecules. Thus we will refer to this state as the ADP/ADP state (Fig 4B). There are no obvious differences when comparing the Rvb1 nucleotide binding pocket in the ADP/ADP (native) dimer and in the ADP/PP (SeMet) dimer (Fig 4). In detail, the β-phosphate of the ADP molecule is bound to the Walker A through Lys77, while Asp405 from sensor II that discriminates between bound and unbound nucleotide interacts with the α-phosphate. In contrast, we observe subtle changes in the case of Rvb2. First, Arg399 in sensor II is interacting with the adenine in the ADP form and bends towards one of the sulphates in the PP state. Second, Asp298 in the Walker B motif flips towards the sulphate in the PP state. Third, Asn328 in sensor I interacts with the β-phosphate and the sulphate in the Rvb2 states (ADP or PP, respectively), while in Rvb1 it remains more distant. Finally, the “trans-arginine finger” Arg358 of Rvb1 that protrudes into the Rvb2 nucleotide binding pocket stabilizes both the ADP and the PP state. No apparent differences between the two nucleotide-binding states were found in the dodecameric assemblies. However, superimpositions of the respective dimers (ADP/ADP and ADP/PP, r.m.s.d._830Cα_ = 0.44 Å) revealed subtle movements in the secondary elements, which surround the Rvb2 catalytic site when bound to ADP or sulphates (Fig 4C). This is further supported by maximum likelihood superimpositions and B-Factor calculations [22,23] that highlight the flexibility around the Rvb2 nucleotide pocket (Fig 4D). The heat map also indicates that in both nucleotide states the linkers in Rvb1 and Rvb2 show the highest mobility in the middle part (residues 128–129 and 231 to 233 in Rvb1; and 236–237 in Rvb2). Interestingly, this region is connected through a well-structured N-terminal tail with the catalytic sites in Rvb1 and Rvb2. Despite some missing residues in the sulphate-bound Rvb2, this tail has a similar position as in the ADP-bound Rvb1 (S4 Fig). We speculate that the N-terminal tail may relay conformational changes between active sites and the DII domains, which were proposed to be flexible by molecular dynamics simulations [5,17]. In summary, despite the described changes in the nucleotide binding pocket there are no significant structural differences between the two Rvb1/Rvb2 dimers (ADP/ADP and ADP/PP), nor in comparison with the ATP/apo dimer [18] that would affect the dodecameric assemblies in the different hydrolysis states.

### A core-core dodecameric conformation is also found in the crystal packing

As an alternative to the dodecameric assembly where interactions between hetero-hexameric rings are mediated by the DII domains of Rvb1 and Rvb2 (DII-DII), a second dodecameric arrangement of the Rvb1/Rvb2 complex is observed in the crystal, where the interface between adjacent hetero-hexameric rings is mediated by the AAA+ core (core-core) (S5A Fig). SAXS data suggested that such a mixture of dodecameric assemblies, mediated by DII-DII and core-core interactions, might also exist in solution [18]. However, dodecameric Rvb1/Rvb2 complexes involving core-core interactions have never been observed in any of the previous EM studies. Moreover, crosslinking mass spectrometry data solely support the DII-DII conformation [18] where the interaction surface is highly conserved (S5B Fig). To solve this discrepancy, we also analysed the *Ct* Rvb1/Rvb2 complex by cryo-EM to test if both arrangements do exist in solution.

### Reference-free 2D analysis revealed asymmetric features in the *Ct* Rvb1/Rvb2 complex

**Fig 5:**
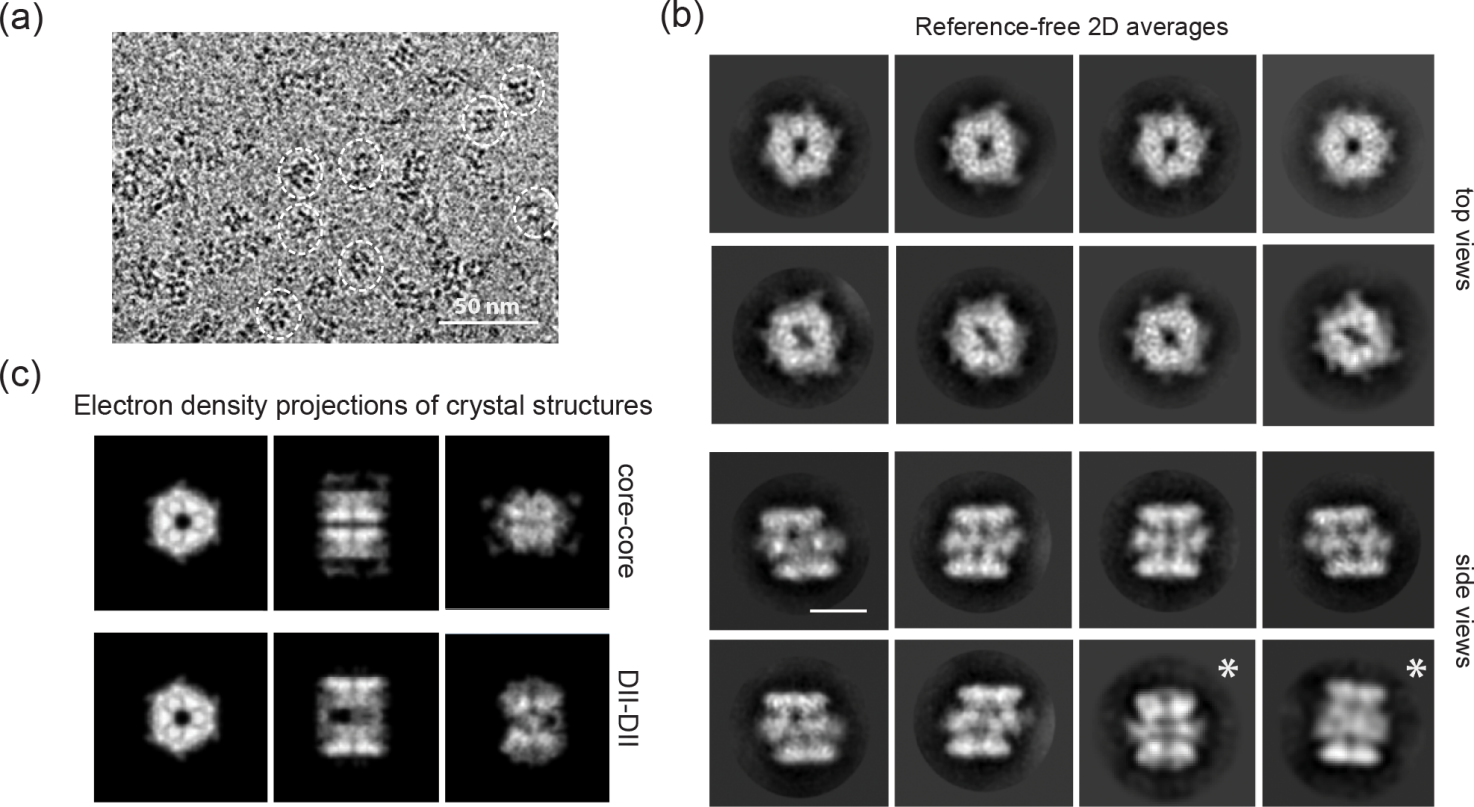
Reference-free 2D analysis of the frozen-hydrated Rvb1/Rvb2 complex. (a) Representative cryo-EM field of the Rvb1/Rvb2 complex. Complexes in side and top views are highlighted. Scale bar, 50 nm. (b) Reference-free 2D averaged images obtained with RELION. Top views are represented in the two upper rows and side views in the two lower rows. The stretched classes are marked with an asterisk. Scale bar, 100 Å. (c) Projections of the electron density of the two possible Rvb1/Rvb2 arrangements in the crystal: core-core and DII-DII.

Freshly purified Rvb1/Rvb2 complex was prepared following exactly the same protocol as for the crystallographic analysis and was analysed using single-particle cryo-EM. The micrograph field in Fig 5A shows typical top and side views. 340,000 particles were automatically selected and two-dimensional (2D) classification was performed in RELION [24,25]. None of the reference-free averaged images (Fig 5B) showed the core-core conformation, as indicated by comparison with the projections of the electron density of the crystal structure (Fig 5C) and by the presence of three layers of density that have been previously described. Accordingly, the two outer electron dense layers correspond to the hetero-hexameric rings in side views, while the middle region comprises the flexible DII domains. Furthermore, the outer layers show high resolution features, while the middle region remains more blurry. In the majority of the side views the complexes are ~130 Å in height, although in a few classes (8% of the side view-particles) the complex has a more stretched shape. We will therefore refer to them as ‘compact’ and ‘stretched’, as previously proposed [7]. Different conformations of the Rvb1/Rvb2 dodecamer have been described before in human and yeast [7,13], and the ratio between them varies substantially between species. However, the abundance of the compact conformation observed in *Chaetomium thermophilum* is in agreement with previous studies in yeast and human [7,13,16]. Moreover, the proportion of stretched conformation in yeast (12%) correlates with the one obtained in *Chaetomium thermophilum* (this study) where only 8% of the side views show a stretched conformation. Interestingly, the majority of the averaged images revealed asymmetric features in the Rvb1/Rvb2 complex such as the slanted rings present in the compact side views and the variable shape of the central channel in the top views (Fig 5B). Unevenly distributed densities can be also distinguished in the periphery of the top view-rings. This asymmetric arrangement appears to contradict the D3 symmetry observed in the crystal structure, suggesting that there are variations in the interaction between the rings in solution. The comparison of the compact 2D averages with the projections of the crystal structure confirms this observation, as none of the projections showed slanted rings or elongated channels (Fig 5C). On the other hand, all the stretched 2D averages showed straight rings and match in height with the projections of the electron density of the crystal structure, although the middle part is slightly different, suggesting a flexible DII position. The fact that the averaged side views show slanted and straight rings raised the question whether the asymmetric features reflects additional conformational states or several views of the same complex. We therefore performed a three dimensional (3D) reconstruction to elucidate how the asymmetry of the complex relates to the different conformational states.

### Characterization of the compact Rvb1/Rvb2 conformation

**Fig 6:**
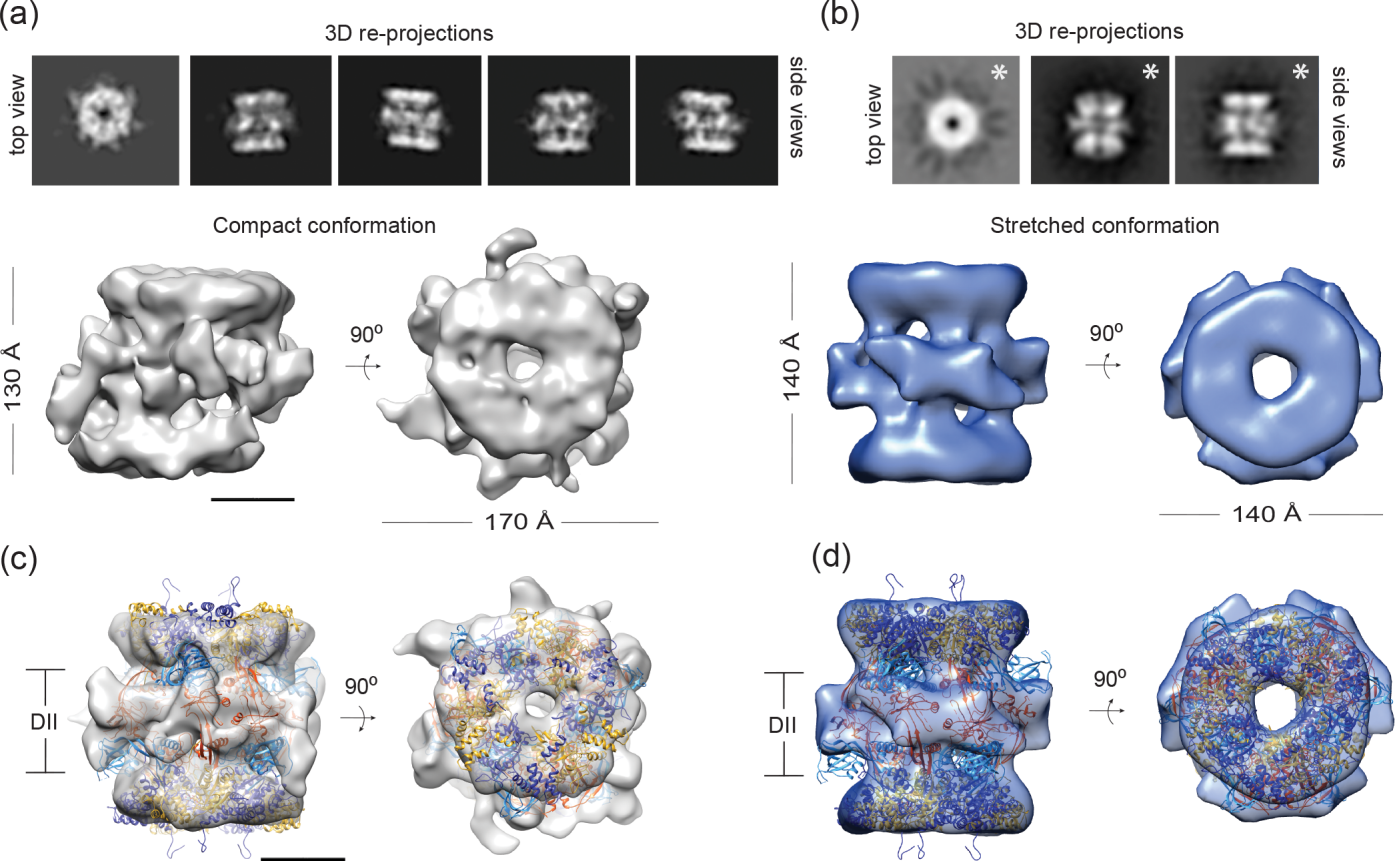
Cryo-EM reconstruction of Rvb1/Rvb2 complex in compact and stretched conformations and fitting of the crystal structure. (a) Re-projection of the compact 3D model (top) and non-symmetrised 3D model of the compact Rvb1/Rvb2 complex at 12 Å resolution in two different views (bottom). Scale bar, 50 Å. (b) Re-projection of the stretched 3D model (top) and D3-symmetrised volume of the stretched Rvb1/Rvb2 complex at 21 Å resolution in two different views (bottom). (c) The crystal structure of the Rvb1/Rvb2 dodecamer (gold/orange and blue/bright blue for Rvb1 and Rvb2 respectively) is fitted into the compact 3D reconstruction in side and top views. (d) The crystal structure of the Rvb1/Rvb2 dodecamer (same colour code) is fitted into the stretched cryo-EM model in side and top views. Scale bar, 50 Å.

The images corresponding to the stretched and compact conformations were split based on the 2D classification, and each dataset was processed independently. Regarding the compact conformation, after 3D classification 33,710 particles were used to generate a non-symmetrised 3D model of the dodecameric Rvb1/Rvb2 complex with an estimated resolution of 12.3 Å (Fig 6A) based on the Fourier Shell Correlation (FSC) = 0.143 criteria [26,27] and 16 Å based on the FSC = 0.5 criteria (S6A Fig). Re-projections of the 3D model (Fig 6A) correspond well with the reference-free 2D class averages (Fig 5B). Straight and slanted rings are present in the re-projections of the compact 3D model confirming that the different degrees of displacement present in the 2D averages could in principle be explained by a single 3D conformation–at least at the given resolution. Although the homogeneity of dodecamers in our purification and the abundance of compact conformation justified the use of top and side views for the reconstruction, we also generate a 3D model only from side views, as previously described [7,13]. The latter converged to an identical 3D model, albeit the resolution was slightly reduced (20 Å) most likely due to the limited number of particles (13541 particles) (S6B Fig). The compact Rvb1/Rvb2 complex is formed by two identical slanted rings that interact in the middle part through unevenly distributed lobes (Fig 6A). It is thus obvious to conclude that the rings would correspond to the core domains while the flexible DII domains localize in the middle part. The dimensions of the complex (~130 Å height, ~170 Å maximum width and ~20 Å central channel) are very similar to the yeast dodecamer previously solved by cryo-EM [16]. The slanted position of the rings becomes apparent when rotating the complex along the longitudinal axis (S6C Fig), and is also evident in the top view, where parts of the bottom ring are also visible (Fig 6A). However, further information is needed regarding the biological function of the slanted rings. The tilted arrangement of two hexameric rings has been observed in other helicases such us the MCM2-7 [28] and the SV40 large tumor antigen [29], both suggesting the implication of the ring offset in the initiation of DNA replication. As the helicase activity of the Rvb1/Rvb2 dodecamer is poorly understood, the role of the slanted rings in the DNA interaction still needs to be studied. We currently favour the idea that the plasticity of the rings within the dodecamer might facilitate the asymmetric interaction with other proteins (e.g. INO80 complex). Interestingly, our cryo-EM complex fits reasonably well into the head domain of the INO80 complex (EMDB-2385), which was assigned to Rvb1/Rvb2 heterododecamer by chemical crosslinking and mass spectrometry [8] (S6D Fig).

### Characterization of the stretched Rvb1/Rvb2 conformation

Stretched side views were further classified in 3D and 1810 particles were used to obtain a D3-symmetrised volume (Fig 6B) at 21.4 and 24.7 Å according to the FSC = 0.143 and FSC = 0.5 criteria, respectively, S7A Fig). Although the 3D models were refined independently with and without symmetry, the re-projections of the D3 symmetrised model correlate better with the stretched reference-free 2D class averages (Fig 6B and S7B Fig). Given the reduced number of particles present in the stretched 3D model, the better correlation may partly be due to imposing additional rotational symmetry. Thus, the stretched models with and without D3 symmetry imposition were compared (S7C Fig) and confirmed that applying D3 symmetry was indeed justified. The stretched Rvb1/Rvb2 complex is formed by two rings, one on top of each other, interacting through uniformly disposed lobes. The dimensions of the complex are 140 Å height, ~140 Å width and a central channel of ~30 Å. The complex resembles the human Rvb1/Rvb2 cryo-EM model [7].

It is worth noting that both 3D models of the Rvb1/Rvb2 complex in the compact and stretched conformation show a DII-DII ring-interface. Hence, the core-core arrangement appears not to be present in solution.

The final resolution of the stretched complex is in accordance with the features shown by the 2D class averages and the limited number of particles in the final model. In contrast, the high resolution features apparent in some of the 2D class averages are missing in the final compact model (Figs 5B and 6A). A recent study in yeast also pointed out that the presence of multiple intermediates in low amounts prevented a high resolution structure determination [13]. We can thus speculate that the presence of conformational transitions, between the stretched and compact conformations, and specially the flexibility of the DII domains may have prevented the alignment to converge to a high resolution structure. This became evident during the 2D classification, where only some of the averaged images showed high resolution features and from those just a fraction had a well-defined DII interface thereby considerably reducing the number of similar particles contributing to the final model. In addition, the well-resolved 2D class averages might represent conformation-specific preferred orientations. Other orientations of these conformations would thus be under-represented and consequently limit the attainable resolution of the 3D structural analysis.

### Fitting of the crystal structure into the cryo-EM reconstruction of the compact and stretched complexes

To further investigate the plasticity of the dodecameric Rvb1/Rvb2 complex, we compared the crystal structure with the EM densities of the compact and stretched complexes. Rigid-body fitting of the crystal structure into the compact conformation shows that the crystal structure is more elongated than the compact cryo-EM model (Fig 6C). Docking of the DI and DIII domains (i.e. the core without the flexible loops), accounts for the ring densities (cam score = 0.5) and highlights the region that would be occupied by the DII domains (S8A Fig). At the resolution attained, the structure of the rings themselves does not vary as compared to the crystal structure. In contrast, displacing one ring with respect to the other would require the rearrangement of domains DII as well as compacting and widening the Rvb1/Rvb2 complex, as seen in solution. However, at the given resolution and considering their reported flexibility, individual positions of the DII domain between the rings cannot be inferred.

In contrast, the Rvb1/Rvb2 crystal structure nicely fits into the stretched cryo-EM reconstruction (Fig 6D). The DI and DIII domains fit well into the rings, suggesting that the transition between compact and stretched conformation does not affect the overall structure of the upper and lowers ring but rather their relative positions. At the same time the Rvb1 DII/DII interface accounts for the protruding lobes of the middle region. This interface seems to be important for dodecamer formation as deletion of the Rvb1 DII domain dramatically reduced the abundance of the dodecamer [6], supporting its stable position in the stretched conformation. In contrast, the Rvb2 DII domains in the crystal structure fold back to the ring and stick out of the EM density of the stretched complex, although unassigned densities in close proximity to the protruding Rvb2 DII β-strands may suggest their position (S8B Fig). Interestingly, crystal packing forces by neighbouring symmetry-related rings constrain the Rvb2 DII position (S5A Fig). Moreover, deletion of Rvb2 DII domain did not significantly affect dodecamer formation [6] suggesting that the Rvb2 DII domains might be quite flexible in solution. In fact, we believe that the intrinsic flexibility of Rvb2 DII accounts for the main rearrangements of the middle plane in the compact and stretched conformations. This conformational flexibility results in changes in the binding surfaces of the dodecamer. Hence, in the context of larger protein assemblies it is likely that Rvb1/Rvb2 functions as an interaction platform interacting with other proteins and nucleic acids presumably mediated through the DII domains.

## Conclusions

Crystal structures of the dodecameric Rvb1/Rvb2 complex from *Chaetomium thermophilum* have been independently determined by two groups in the ADP/ADP and ADP/PP states at 2.9 Å and 3.0 Å, respectively (this study) and in the ADP/ADP and ATP/apo states at 2.9 and 3.6 Å, respectively [18]. The four structures are almost identical, with r.m.s. deviations between 0.9 and 1.1 Å. Based on their SAXS experiments, Lakomek et al. suggested that Rvb1/Rvb2 dodecamers form a mixture in solution where interactions between the two hetero-hexameric rings are mediated by DII-DII and core-core interactions, while complementary cross-linking data only supported the DII-DII interactions. In the present study we have assessed the Rvb1/Rvb2 complex by cryo-EM and only observe Rvb1/Rvb2 dodecamers with DII-DII interactions. The inherent conformational variability of the Rvb1/Rvb2 complex in solution possibly accounts for the differences between the X-ray and cryo-EM structures using the same sample, but also for our difficulties determining cryo-EM structures of the Rvb1/Rvb2 dodecamer at high resolution. The transition between the compact and the stretched conformation presumably implicates conformational changes of the DII domains of Rvb1 and Rvb2. Intermediate conformational states have been observed in yeast [13], and DII flexibility has been previously suggested [5,17]. The crystal structure and a minor fraction of the cryo-EM particles show the Rvb1/Rvb2 complex in a stretched conformation suggesting that the D3 symmetry and the Rvb1 interface are preserved in the crystal and in solution. In contrast, the positions of the Rvb2 DII domains observed in the crystal have not been observed in any of the EM reconstructions up to date. Apparently, the stretched conformation does not represent a major conformation in solution and only represents a sparsely populated class under cryo-EM conditions. Instead, the main state in solution is the compact conformation resulting from an asymmetric reorganization of the DII domains. This novel arrangement with two slanted rings unevenly interacting via the DII domains may contribute to the reported variety of interactions essential for the multiple roles of Rvb1/Rvb2 inside the cell.

## Materials and Methods

### Cloning, expression and purification of *Ct* Rvb1 and Rvb2

*Ct Rvb1* and *Rvb2* genes were identified following recently described procedures [30]. Full-length ORFs of Rvb1 (residues 1 to 462) and Rvb2 (residues 1 to 488) were amplified from cDNA and inserted into the pET-MCN vector that contains an amino terminal, tobacco etch virus (TEV) cleavable histidine tag. Rvb1 and Rvb2 proteins were individually overexpressed in *E*. *coli* BL21 (DE3) Star pRARE strain. For the preparation of native proteins, cells were grown in terrific broth (TB) medium while cells expressing selenomethionine (SeMet) were grown in M9 medium supplemented with 100 mg lysine, threonine, and phenylalanine and 50 mg valine, leucine, isoleucine and seleonomethionine per liter. Protein expression was induced at OD_600_ = 0.6 by addition of 0.7 mM IPTG. Following induction cells grew at 18°C for 12–15 hours before harvesting. Subsequently, the cells were lysed using a french press in 50 mM Tris-HCl pH 7.5, 300 mM NaCl, 10 mM imidazole, 5% glycerol, 2 mM 2-mercaptoethanol, 1 mg/ml DNase and protease inhibitors (Roche). The soluble fraction was cleared by centrifugation (20,000 g for 45 min at 4°C). The 6-His-tagged proteins were purified using nickel (Ni) NTA agarose (Quiagen) affinity chromatography, 350 mM imidazole was used to elute the proteins. After cleavage of the His-tag a second Ni-affinity step was applied to remove TEV protease. The final purification step comprises size exclusion chromatography (SEC) using a Superdex 200 column (GE Healthcare) in 20 mM Tris-HCl pH 7.5, 150 mM NaCl and 2 mM 2-mercaptoethanol. To reconstitute the complex, Rvb1 and Rvb2 proteins were incubated at 4°C for 30 min at 1:1 molar ratio and purified using SEC on a Superose 6 (GE Healthcare) in 20 mM Tris-HCl pH 7.5, 150 mM NaCl, 8% glycerol, 2 mM 2-mercaptoethanol and 5 mM MgCl_2_. An analytical Superdex 200 column (GE Healthcare) was used to study the oligomeric state of the individual proteins and the Rvb1/Rvb2 complex.

### Circular dichroism spectroscopy and heat denaturation

Thermostability of individual proteins and the Rvb1/Rvb2 complex was measured using a CD spectrometer (J-815 Jasco). Initial CD spectra were recorded at concentrations of 0.5–0.7 g/l from 199 to 250 nm using a 1 mm path-length quartz cuvette. For thermal denaturation experiments the temperature was raised from 5°C to 90°C in steps of 1°C per minute, while the CD signal was recorded at 222 nm.

### Crystallization and X-ray structure determination

Full-length Rvb1/Rvb2 complex at a concentration of 6 mg/ml was tested using commercial crystallization screens. The best crystals grew within approximately 5 to 10 days using the hanging-drop method. The optimized crystallization condition contained 0.1 M Bis-Tris pH 5.7 with 1.8 M ammonium sulphate for the ADP/PP state (SeMet) and 0.1 M MES pH 6.0, 1 M succinate, 1.2% MME as precipitant for the ADP/ADP (Native) crystal form. ADP was added during the purification and for co-crystallization. The crystals were cryo-protected in mother liquor supplemented with 30% glycerol and flash-cooled in liquid nitrogen. SeMet and native data sets were collected to 3 Å and 2.9 Å resolution, respectively, at ESRF beamline ID-29, integrated and scaled using XDS [31]. SAD phases were calculated from 21 selenium sites (9 sites in Rvb1 and 12 sites in Rvb2 (S3 Fig) using autoSHARP [32] and further improved using solvent flattening. Selenium sites were used as sequence markers to construct the initial model that was initially refined by PHENIX [33] followed by cycles of manual rebuilding in Coot [34] and refinement with BUSTER [35]. For the native Rvb1/Rvb2 complex structure the SeMet complex (ADP/PP state) was used as a starting model and the refinement process was carried out using PHENIX. Data collection and refinement statistics are shown in S1 Table. All structures possess an excellent geometry according to MolProbity [36]. Model figures and superimpositions were prepared using Coot and PyMOL [37]. In the case of the dimer (Fig 4), the maximum likelihood super-positioning and subsequent B-Factor calculation was perfomed using the Theseus program [22,23].

### Cryo-EM and image processing

2.5 μl aliquots of freshly purified complex (20 mM Tris-HCl pH 7.5, 125 mM NaCl, 2 mM 2-mercaptoethanol and 5 mM MgCl_2_) at a concentration of 0.35 mg/ml were incubated for 15 sec on glow-discharged molybdenum grids (400 mesh, 1.2/1.5, Quantifoil). Grids were blotted for 9 sec in 95% ambient humidity and flash frozen in liquid ethane using a FEI Vitrobot. Grids were transferred to a FEI Titan Krios electron microscope that was operated at 300 kV. Images were automatically recorded on a direct electron detector FEI Falcon II at a nominal magnification of 75000, yielding a pixel size of 1.084 Å. The exposure time was 1.28 sec, the total dose was 40 e/Å^2^ (5.7 e/Å^2^ per frame) and 7 frames were acquired. We used frames to apply micrograph-based motion correction with the tool motioncorr [38]. For the final reconstruction motion-corrected sums were used. Micrographs that showed signs of astigmatism or drift were discarded. Defocus values in the final data set ranged from 0.8 to 4.2 μm.

Using EMAN2 [39] ~6000 particles were manually picked and reference-free classification was performed. The two best classes were used as templates for RELION autopicking, where 369918 particles were selected from 1629 micrographs. Contrast transfer function parameters were estimated using CTFFIND3 [40]. 2D and 3D classifications, ‘gold-standard’ refinement and post-processing were performed using RELION. The reference-free 2D class averaging allowed us to discard bad particles and to split the images that correspond to the two conformations of the complex. Each dataset was processed independently.

For the compact conformation we initiated the 3D classification with 340169 particles by using three different initial references: the DII-DII dodecameric crystal structure, the core-core dodecamer and the cryo-EM reconstruction of human Rvb1/Rvb2 (EMDB-2163); all of them low-pass filtered to 60 Å. The first round of 3D classification yielded one good class containing 89931 particles that was then subjected to local 3D classification. The ‘gold-standard’ refinement yielded a final reconstruction at 12.3 Å resolution (33710 particles), based on the FSC = 0.143 criterion and 16 Å according to the FSC = 0.5 threshold (S6A Fig). From those 33710 particles included in the final compact model, all the side views (13541 particles) were refined independently yielding a 20 Å side-views 3D model. For the comparison between the compact final model and the side views model (S6B Fig) the former was low-pass filtered to 20 Å using SPIDER software [41]. The proportion of side views particles in the final model (40%) is the same as the proportion of side views present in the initial dataset (42%).

For the stretched conformation we initiated two independent the 3D classifications with different initial references: the crystal structure the DII-DII dodecameric crystal structure and the cryo-EM reconstruction of human Rvb1/Rvb2 (EMDB-2163); all of them low-pass filtered to 60 Å. 3D classification of 3000 particles yielded a good class of 1810 particles, which were used for the final reconstruction. The ‘gold-standard’ refinement of the stretched 3D model was performed independently with and without D3 symmetry imposed. The D3-symmetrised final volume has 21.4 Å resolution, based on the FSC = 0.143 criterion and 24.7 Å according to the FSC = 0.5 threshold (S7A Fig).

For the projections of the electron density of the crystal structure, the DII-DII and core-core atomic models were first converted into an electron density at 12 Å (the compact model resolution) using Chimera [42]. Secondly, they were scaled and padded to match the voxel size and Å/pixel of the cryo-EM models using EMAN. Finally, the projection library was created using XMIPP [43]. The re-projections of the cryo-EM models were obtained using the ‘create projection library’ tool in XMIPP.

The DII-DII atomic model was automatically fitted as a rigid-body into the two cryo-EM electron densities using Chimera. Fitting of the core domains of Rvb1/Rvb2 hexamers (without the loops) to the compact EM model was performed with FitMap tool of UCSF Chimera in global search mode, using a normalized cross-correlation coefficient as a fit quality measure. The cryo-EM reconstruction of Rvb1/Rvb2 was manually fitted into the INO80 negative stain 3D model (EMDB-2385) using Chimera.

### Accession Numbers

The atomic coordinates and structure factors have been deposited in the Protein Data Bank (http://wwpdb.org/) under accession codes 5FM6 and 5FM7 for Rvb1/Rvb2 ADP/PP and Rvb1/Rvb2 ADP/ADP, respectively. The EM maps have been deposited in the EM Data Bank (http://www.ebi.ac.uk/pdbe/emdb/) with accession numbers EMD-3242 for the compact 3D model and EMD-3243 for the stretched 3D model.

## Supporting Information

S1 Fig Sequence conservation of *Ct* Rvb1 and Rvb2. (a) Sequence alignment of Rvb1 from Chaetomium thermophilum (Ct) with Rvb1 from Saccharomyces cerevisiae and Homo sapiens (Human). (b) Sequence alignment of Ct Rvb2 with Rvb2 from Saccharomyces cerevisiae and Homo sapiens (Human). Identical amino acids are highlighted in gold for Rvb1 and in blue for Rvb2. Rvb1/Rvb2 domains and ATP binding and hydrolysis motifs, Walker A and B, sensor I and II, and arginine finger are indicated.
(TIF)

S2 Fig Absence of Rvb2 hexamers in the Rvb1/Rvb2 complex purification. (a) Representative negatively stained EM field of purified Rvb2 fraction used to generate Ct Rvb1/Rvb2 dodecamer showing that Rvb2 is monomeric. (b) Negative stain EM field of the purified Rvb1/Rvb2 sample in which no single-layer side views (hexamers) are visible
(TIF)

S3 Fig Selenium methionine markers in Ct Rvb1 and Rvb2. (a) Sequence alignment between Rvb1 and Rvb2. The residue numbers for methionines present in Rvb1 and Rvb2 are label in gold and blue, respectively; sequence identity is highlighted in green. Rvb1 and Rvb2 share 42% sequence identity and 63% homology. (b) Silver-stained SDS-PAGE of the dissolved crystal showing the presence of Rvb1 and Rvb2 within the crystal. (c) Ribbon representation of Rvb1 and Rvb2 monomers with anomalous difference Fourier showing the selenomethionine positions.
(TIF)

S4 Fig The well-structured N terminal tail connects the nucleotide binding pocket and the DII domains. (a) ADP-bound Rvb1 (left) and ADP-bound Rvb2 (right) monomers are shown in ribbon representation. The catalytic motifs are depicted in stick representation with Walker A (light green), Walker B (dark green), sensor I (violet) and sensor II (purple). The electron density of the nucleotides and the N-terminal tail are shown in mesh. (b) ADP-bound Rvb1 (left) and PP-bound Rvb2 (right) monomers are shown in ribbon representation. Same colour code as in (a).
(TIF)

S5 Fig Analysis of two possible Rvb1/Rvb2 arrangements in the crystal and surface conservation. (a) The crystal packing shows two different dodecameric arrangements, one where the DII are present in the middle part (DII-DII) and an alternative arrangement where the interaction is mediated by the core domains (core-core). (b) Differential amino acid conservation of the two alternative dimer interfaces. Conserved amino acids are shown in pink and variable residues in blue, both were generated with ConSurf. At the left, the Rvb1/Rvb2 dimer is depicted in ribbons representation with Rvb1 (gold) and Rvb2 (blue).
(TIF)

S6 Fig Cryo-EM analysis of the compact Rvb1/Rvb2 conformation. (a) Fourier Shell Correlation (FSC) curve for the non-symmetrised compact 3D model. The red line indicates FSC = 0.143 and the green line shows FSC = 0.5. (b) Comparison between the compact final model (in grey) and the model generated with the side view images of the final dataset (in pink). The final model was low-pass filtered to the resolution of the side views model for comparison. Scale bar, 50 Å. (c) Non-symmetrised 3D model of the compact Rvb1/Rvb2 complex in two different views. The two views illustrate that the ring displacement changes from 13 to 0 degrees when rotating the complex along the longitudinal axis. (d) Fitting of the 3D model of the compact Ct Rvb1/Rvb2 (grey mesh) into the head domain of the yeast INO80 complex EM reconstruction (EMDB-2385) shown in cyan in two related views. Scale bar, 50 Å.
(TIF)

S7 Fig Cryo-EM analysis of the stretched Rvb1/Rvb2 conformation. (a) Fourier Shell Correlation curve for the D3-symmetrised stretched volume. The red line indicates FSC = 0.143 and the green line shows FSC = 0.5. (b) Comparison between the stretched reference-free 2D averages (upper row), the re-projections of the D3-symmetrised stretched model (middle row) and the re-projections of the non-symmetrised stretched model (lower row). (c) Comparison between the D3 symmetrised (in blue) and the non-symmetrised (in bright blue) stretched 3D models in side and top views. The scale bar corresponds to 50 Å.
(TIF)

S8 Fig Fitting of the crystal structure into the stretched and compact cryo-EM models. (a) Fitting of the AAA+ core (without loops) of the Rvb1/Rvb2 atomic structure (gold and blue for Rvb1 and Rvb2 respectively) into the compact 3D reconstruction in side and top views. Scale bar, 50 Å. (b) The atomic structure is docked into the translucent stretched cryo-EM model in side view to illustrate the unassigned densities that would accommodate the Rvb2 DII domains.
(TIF)

S1 Table Crystallographic Statistics. (DOCX)
