# Supplementary figures and images for: The Combination of X-Ray Crystallography and Cryo-Electron Microscopy Provides Insight into the Overall Architecture of the Dodecameric Rvb1/Rvb2 Complex
Source: PLoS One. 2016 Jan 8;11(1):e0146457. doi: 10.1371/journal.pone.0146457 (PMC4706439; doi:10.1371/journal.pone.0146457)

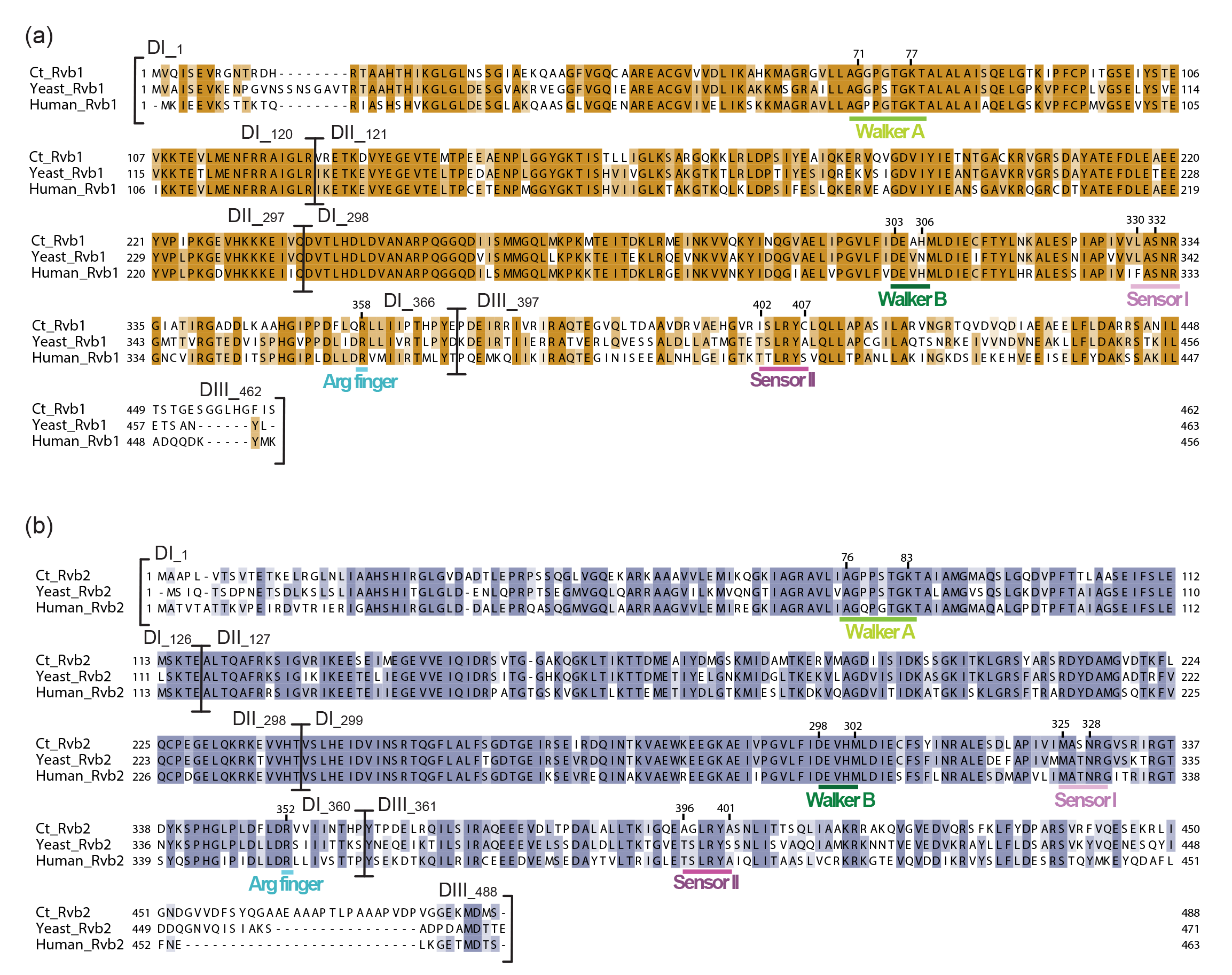

Supplement: S1 Fig — (a) Sequence alignment of Rvb1 from Chaetomium thermophilum (Ct) with Rvb1 from Saccharomyces cerevisiae and Homo sapiens (Human). (b) Sequence alignment of Ct Rvb2 with Rvb2 from Saccharomyces cerevisiae and Homo sapiens (Human). Identical amino acids are highlighted in gold for Rvb1 and in blue for Rvb2. Rvb1/Rvb2 domains and ATP binding and hydrolysis motifs, Walker A and B, sensor I and II, and arginine finger are indicated. (TIF) [file pone.0146457.s001.tif]

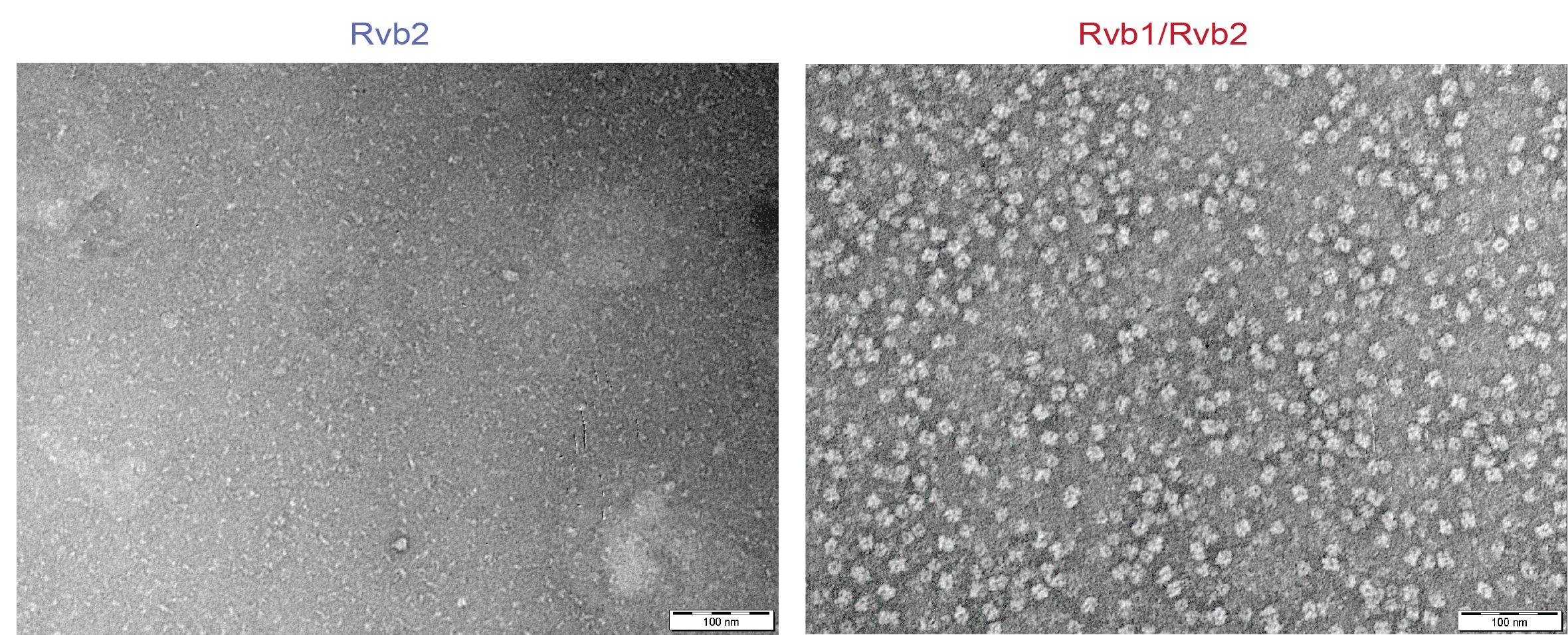

Supplement: S2 Fig — (a) Representative negatively stained EM field of purified Rvb2 fraction used to generate Ct Rvb1/Rvb2 dodecamer showing that Rvb2 is monomeric. (b) Negative stain EM field of the purified Rvb1/Rvb2 sample in which no single-layer side views (hexamers) are visible (TIF) [file pone.0146457.s002.tif]

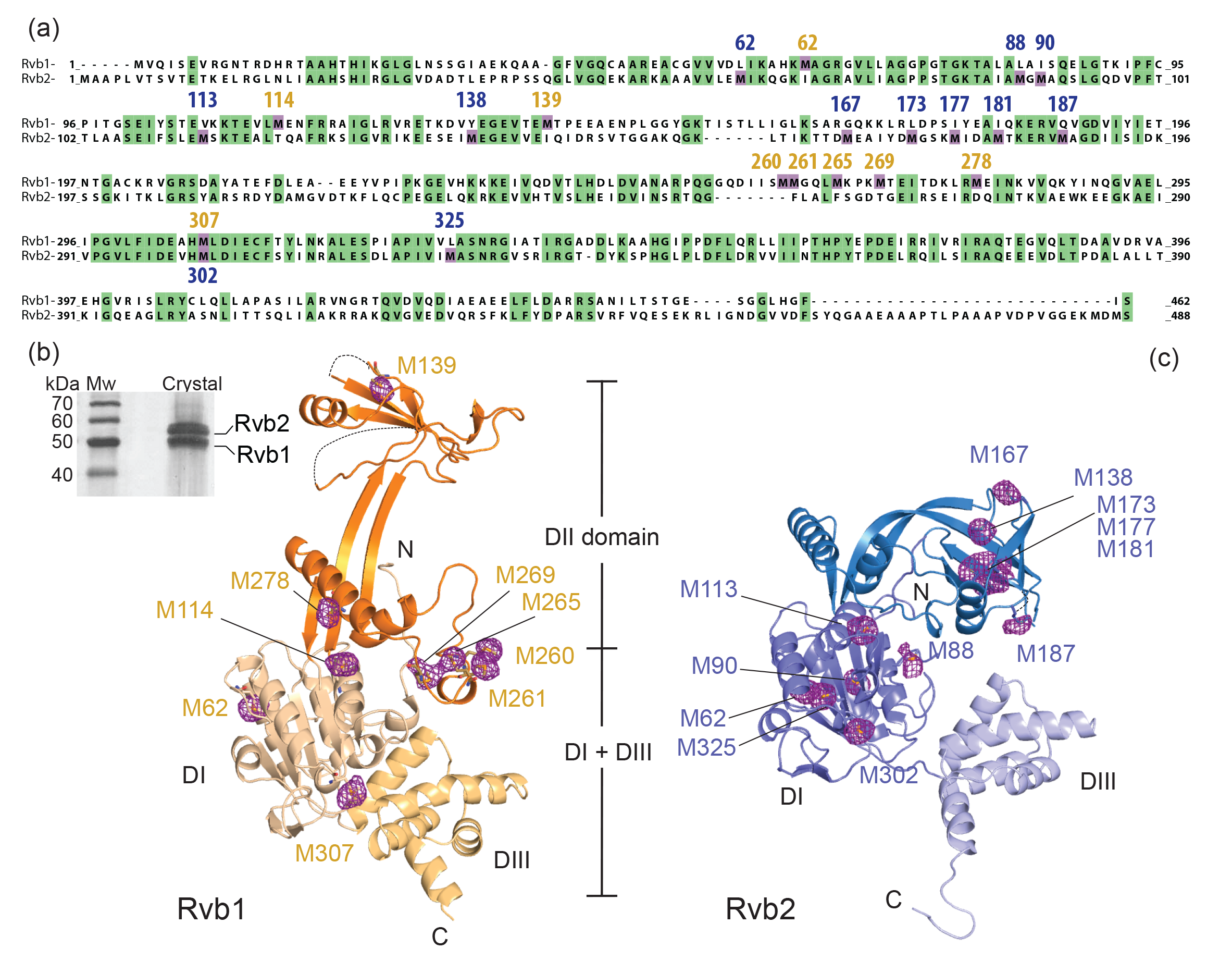

Supplement: S3 Fig — (a) Sequence alignment between Rvb1 and Rvb2. The residue numbers for methionines present in Rvb1 and Rvb2 are label in gold and blue, respectively; sequence identity is highlighted in green. Rvb1 and Rvb2 share 42% sequence identity and 63% homology. (b) Silver-stained SDS-PAGE of the dissolved crystal showing the presence of Rvb1 and Rvb2 within the crystal. (c) Ribbon representation of Rvb1 and Rvb2 monomers with anomalous difference Fourier showing the selenomethionine positions. (TIF) [file pone.0146457.s003.tif]

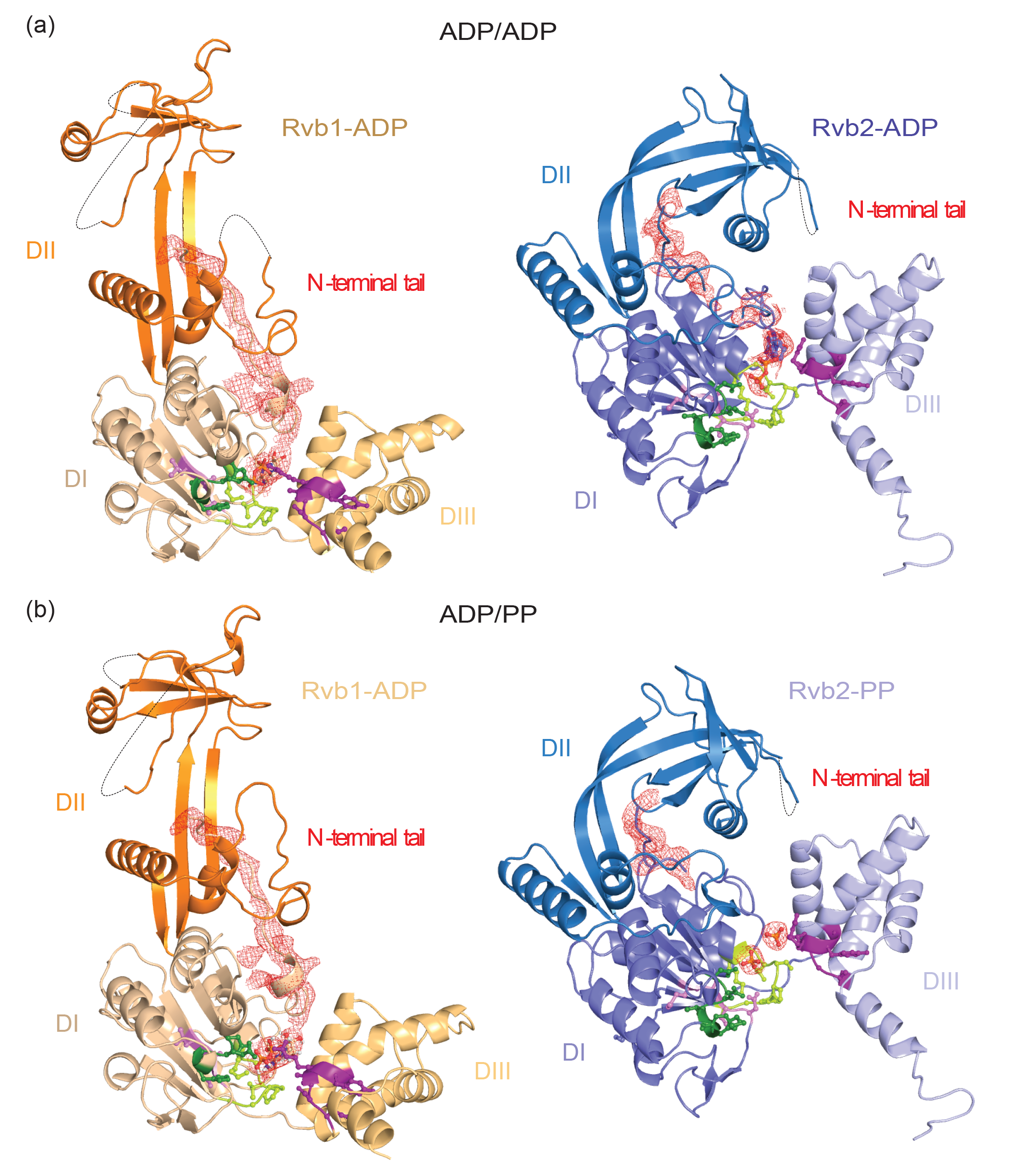

Supplement: S4 Fig — (a) ADP-bound Rvb1 (left) and ADP-bound Rvb2 (right) monomers are shown in ribbon representation. The catalytic motifs are depicted in stick representation with Walker A (light green), Walker B (dark green), sensor I (violet) and sensor II (purple). The electron density of the nucleotides and the N-terminal tail are shown in mesh. (b) ADP-bound Rvb1 (left) and PP-bound Rvb2 (right) monomers are shown in ribbon representation. Same colour code as in (a). (TIF) [file pone.0146457.s004.tif]

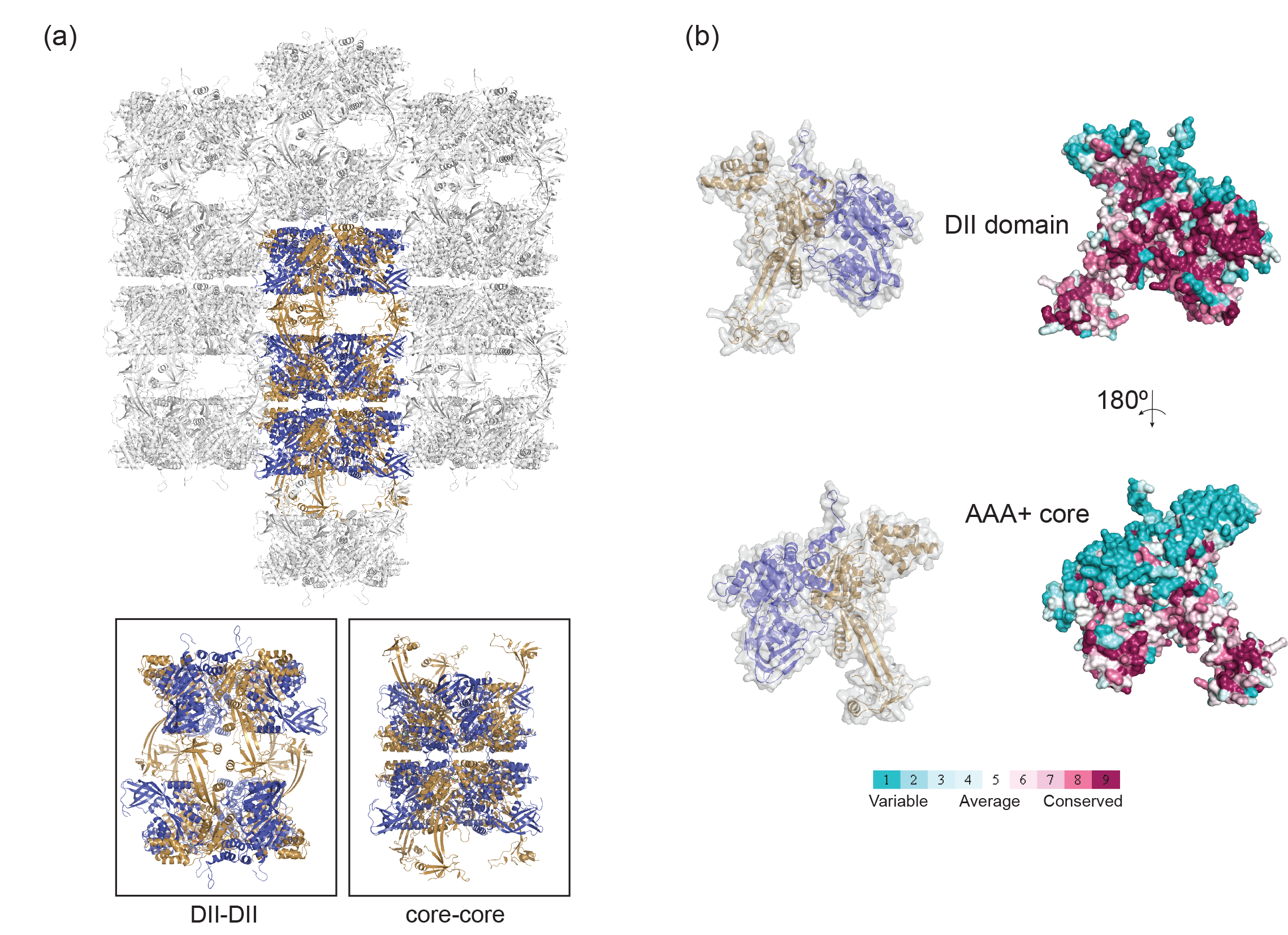

Supplement: S5 Fig — (a) The crystal packing shows two different dodecameric arrangements, one where the DII are present in the middle part (DII-DII) and an alternative arrangement where the interaction is mediated by the core domains (core-core). (b) Differential amino acid conservation of the two alternative dimer interfaces. Conserved amino acids are shown in pink and variable residues in blue, both were generated with ConSurf. At the left, the Rvb1/Rvb2 dimer is depicted in ribbons representation with Rvb1 (gold) and Rvb2 (blue). (TIF) [file pone.0146457.s005.tif]

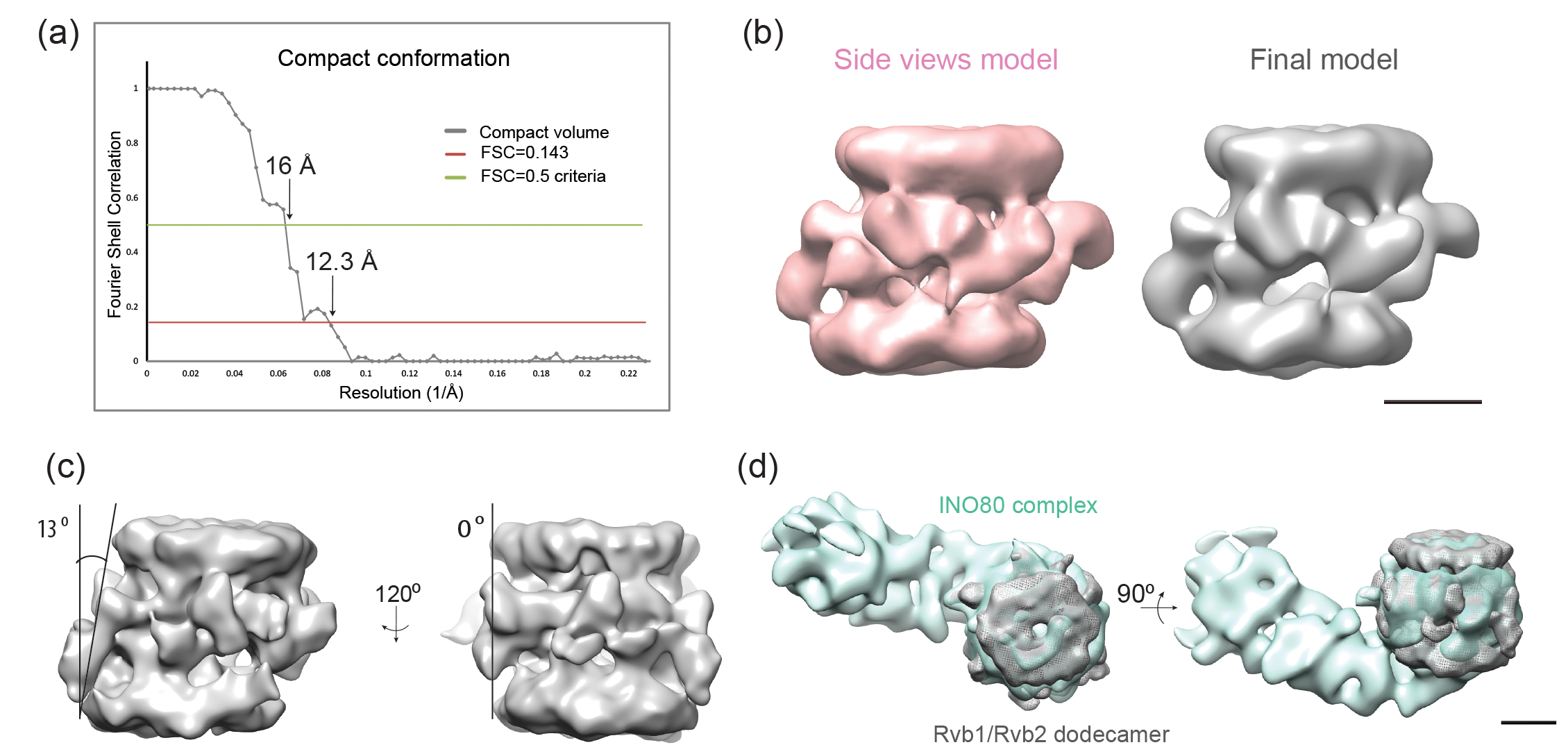

Supplement: S6 Fig — (a) Fourier Shell Correlation (FSC) curve for the non-symmetrised compact 3D model. The red line indicates FSC = 0.143 and the green line shows FSC = 0.5. (b) Comparison between the compact final model (in grey) and the model generated with the side view images of the final dataset (in pink). The final model was low-pass filtered to the resolution of the side views model for comparison. Scale bar, 50 Å. (c) Non-symmetrised 3D model of the compact Rvb1/Rvb2 complex in two different views. The two views illustrate that the ring displacement changes from 13 to 0 degrees when rotating the complex along the longitudinal axis. (d) Fitting of the 3D model of the compact Ct Rvb1/Rvb2 (grey mesh) into the head domain of the yeast INO80 complex EM reconstruction (EMDB-2385) shown in cyan in two related views. Scale bar, 50 Å. (TIF) [file pone.0146457.s006.tif]

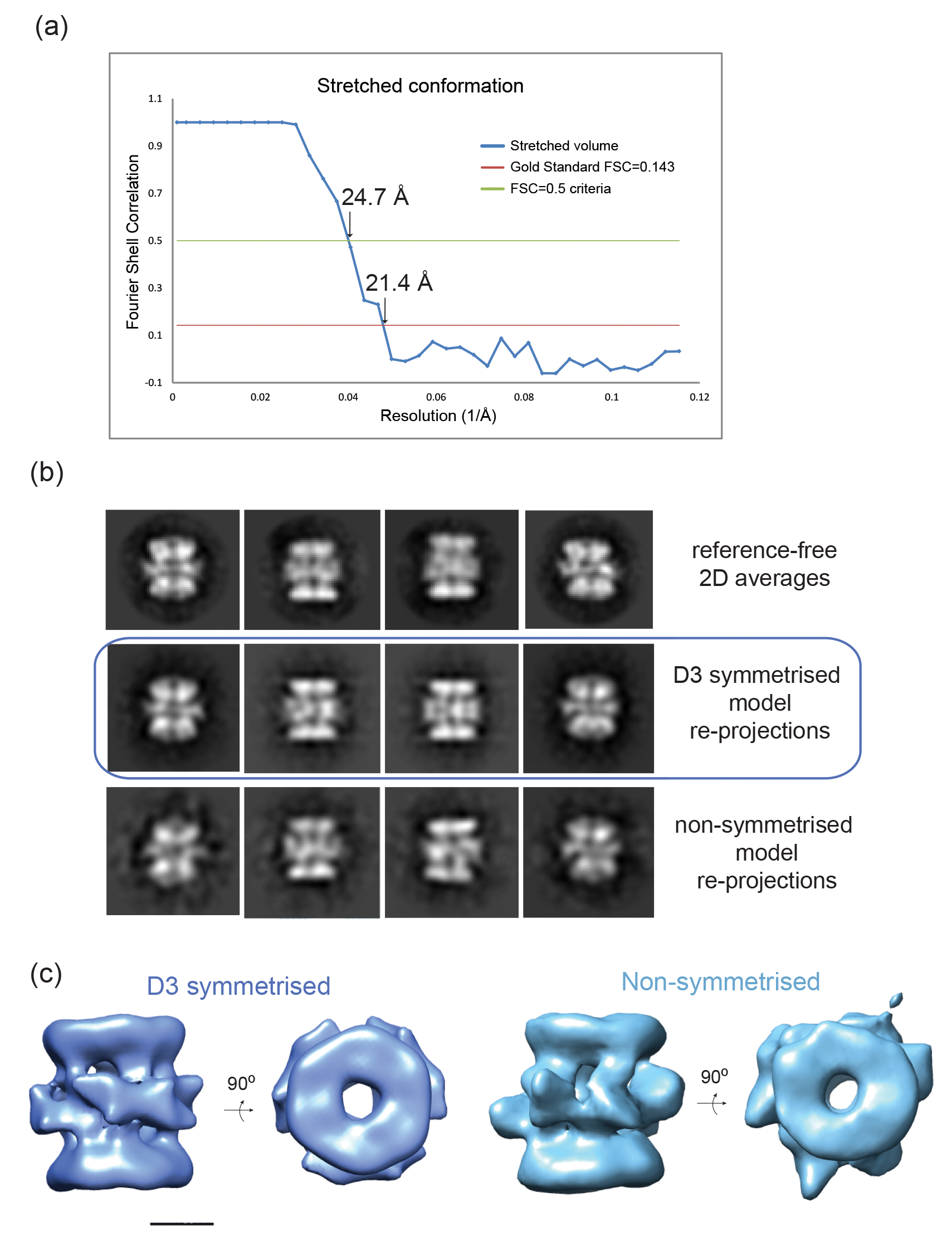

Supplement: S7 Fig — (a) Fourier Shell Correlation curve for the D3-symmetrised stretched volume. The red line indicates FSC = 0.143 and the green line shows FSC = 0.5. (b) Comparison between the stretched reference-free 2D averages (upper row), the re-projections of the D3-symmetrised stretched model (middle row) and the re-projections of the non-symmetrised stretched model (lower row). (c) Comparison between the D3 symmetrised (in blue) and the non-symmetrised (in bright blue) stretched 3D models in side and top views. The scale bar corresponds to 50 Å. (TIF) [file pone.0146457.s007.tif]

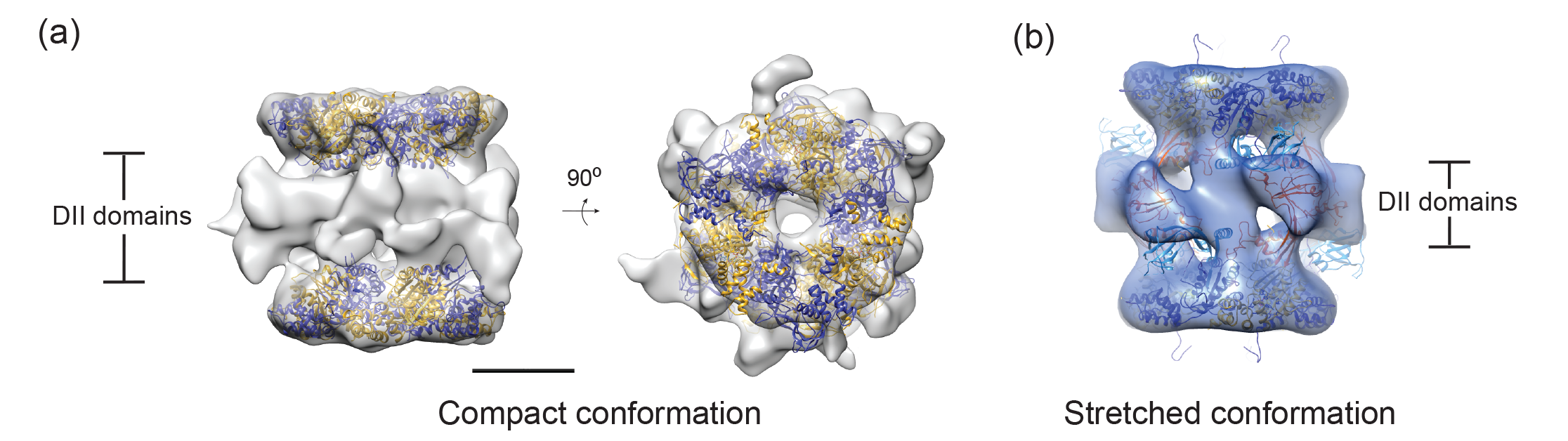

Supplement: S8 Fig — (a) Fitting of the AAA+ core (without loops) of the Rvb1/Rvb2 atomic structure (gold and blue for Rvb1 and Rvb2 respectively) into the compact 3D reconstruction in side and top views. Scale bar, 50 Å. (b) The atomic structure is docked into the translucent stretched cryo-EM model in side view to illustrate the unassigned densities that would accommodate the Rvb2 DII domains. (TIF) [file pone.0146457.s008.tif]
