# Supplementary material for: The Combination of X-Ray Crystallography and Cryo-Electron Microscopy Provides Insight into the Overall Architecture of the Dodecameric Rvb1/Rvb2 Complex
Source: PLoS One. 2016 Jan 8;11(1):e0146457. doi: 10.1371/journal.pone.0146457 (PMC4706439; doi:10.1371/journal.pone.0146457)
Supplement: S1 Table — (DOCX) [file pone.0146457.s009.docx]

**S1 Table.** **Crystallographic Statistics**

|  | Rvb1/2 SeMet  ADP/PP | Rvb1/2  ADP/ADP |
| --- | --- | --- |
| **Data collection** |  |  |
| Beamline | ID29 (ESRF) | ID29 (ESRF) |
| Space group | H32 | H32 |
| Cell dimensions  *a*, *b*, *c* (Å) | 209.61 209.61 137.90 | 208.77 208.77 138.45 |
|  |  |  |
| Wavelength (Å) | 0.97923 | 0.97239 |
| Resolution (Å)^1^ | 50-3.0 (3.08-3.0) | 50–2.9 (2.98-2.9) |
| CC(1/2) | 0.99 (0.53) | 0.99 (0.41) |
| R_merge_ (%) | 11.2 (190.5) | 11.6 (234.4) |
| I/σI | 15.4 (1.2) | 11.5 (0.8) |
| Completeness (%) | 99.9 (98.8) | 100.0 (99.9) |
| Redundancy | 9.8 (9.3) | 10.3 (10.6) |
| Sites | 21 Se |  |
|  |  |  |
| **Refinement** |  |  |
| Resolution (Å) | 50 - 3.0 | 50 - 2.9 |
| No. reflections | 45327 | 25670 |
| R_work_/ R_free_ (%) | 22.99/25.61 | 25.86/28.22 |
| No. atoms |  |  |
| Protein | 6521 | 6486 |
| Ligand | 37 | 54 |
| Water | 0 | 0 |
| B-factors (Å^2^) |  |  |
| Protein | 104.8 | 127.5 |
| Ligand | 90.5 | 120.8 |
|  |  |  |
| R.m.s deviations |  |  |
| Bond lengths (Å) | 0.003 | 0.002 |
| Bond angles (º) | 0.582 | 0.614 |

^1^ Values in parentheses correspond to the highest-resolution shell.
